# Pakistan’s Response to COVID-19: Overcoming National and International Hypes to Fight the Pandemic

**DOI:** 10.2196/28517

**Published:** 2021-05-19

**Authors:** Hashaam Akhtar, Maham Afridi, Samar Akhtar, Hamaad Ahmad, Sabahat Ali, Sundas Khalid, Sajid Mahmood Awan, Shahzaib Jahangiri, Yousef Saleh Khader

**Affiliations:** 1 Yusra Institute of Pharmaceutical Sciences Yusra Medical and Dental College Islamabad Pakistan; 2 Department of Biotechnology Quaid-i-Azam University Islamabad Pakistan; 3 Department of Gynecology and Obstetrics Paf Hospital Islamabad Pakistan; 4 School of Chemical and Materials Engineering National University of Science and Technology Islamabad Pakistan; 5 Medical Education and Biostatistics Department of Community Medicine, Public Health and Family Medicine, Faculty of Medicine Jordan University of Science and Technology Irbid Jordan

**Keywords:** community health, coronavirus, COVID-19, epidemic, epidemiology, Pakistan, pandemic, public health emergency of international concern

## Abstract

The COVID-19 outbreak started as pneumonia in December 2019 in Wuhan, China. The subsequent pandemic was declared as the sixth public health emergency of international concern on January 30, 2020, by the World Health Organization. Pakistan could be a potential hotspot for COVID-19 owing to its high population of 204.65 million and its struggling health care and economic systems. Pakistan was able to tackle the challenge with relatively mild repercussions. The present analysis has been conducted to highlight the situation of the disease in Pakistan in 2020 and the measures taken by various stakeholders coupled with support from the community to abate the risk of catastrophic spread of the virus.

## Introduction

COVID-19 started as an outbreak of a series of unusual pneumonia cases in Wuhan, China, with the earliest cases reported to the World Health Organization’s country office in China on December 31, 2019. By January 12, 2020, the causative agent was identified as a novel coronavirus, initially termed “2019-nCoV,” and up to 41 cases had been preliminarily diagnosed [1,2]. The virus has since been renamed as SARS-CoV-2 and spread drastically, and COVID-19 was declared as pandemic by the World Health Organization (WHO) on March 11, 2020 [3]. Having currently affected more than 210 countries and territories, with 19,550,650 active cases and a mortality rate of 2.85% as of December 31, 2020, the COVID-19 pandemic continues to be a major global public health concern [4].

## Pakistan: Epidemiologic Profile

COVID-19 cases were reported from Islamabad and Karachi on February 26, 2020 [5]. Pakistan being one of the most densely populated countries in Asia, with a population of 204.65 million, and Karachi being the largest metropolitan city in Pakistan, has been greatly vulnerable to this outbreak [6]. Owing to its present economic condition, health care resources, and the occurrence of previous outbreaks, the Centers for Disease Control and Prevention had already issued a level 3 warning for international travelers to Pakistan [7]. The administration had a huge responsibility to constrain the spread through a timely response and the adoption of appropriate measures to avoid any major catastrophe. The disease was initially difficult to contain, especially because of noncompliance of the general population to the necessary measures and timely reporting of symptoms. Within 45 days, on April 10, 2020, Pakistan reported 4601 confirmed cases with a death toll approaching 66 individuals [8].

## Public and Community Response to the COVID-19 Pandemic

There was a mixed response from the community. Few people paid attention to the news and some even considered it fake. News of the virus being a hoax or propaganda spread greatly worldwide and countered the efforts of governments and other agencies that made marked efforts to tackle the spread of the pandemic [9,10]. Common rumors on COVID-19 emergence and treatment through the media and social media resources are presented in Table 1.

**Table 1:** Rumors related to COVID-19 emergence and treatment.

Rumors	Source
The virus is no worse than the one that causes common cold	WHO’s COVID-19 advice for the public: Mythbusters [9]
Hand dryers are effective in eliminating the coronavirus	Chinese social media pages [9,10]
Coronavirus originated with people eating bats in China	YouTube videos, conservative tabloids, blogs, and Twitter [9,10]
There is a vaccine or cure for coronavirus, which the government will not release	Facebook post containing a screenshot of a patent CO-V vaccine by the Centers for Disease Control and Prevention on January 22, 2020 [9,10]
Coronavirus will disappear by the summer	WHO’s COVID-19 advice for the public: Mythbusters [9,11]
Vitamin C can help you ward off coronavirus	A popular social media post [12]
A “miracle” bleach product can cure coronavirus	Far-right conspiracy theory QAnon misinforming people to drink Miracle Mineral Solution, a bleach-based product [13]
Dean Koontz predicted the coronavirus in his 1981 novel *The Eyes of Darkness*	Dean Koontz’s 1981 novel *The Eyes of Darkness* became very popular on Twitter [10]
Osaka flu shown in the television show The Simpsons	Screenshots allegedly from the “Marge in Chains” episode in 1993 on social media [10]
If you cannot hold your breath for 10 seconds without coughing, then you have coronavirus	Self-check coronavirus tests on social media originating in March 2020 [10]
The country will be placed in a nationwide quarantine effective immediately	Text messages claiming the implementation of a country-wide lockdown [10]

## Government’s Initiatives to Tackle the Pandemic

The government of Pakistan has been lauded by international organizations including the WHO (and rightly so) for taking the necessary precautions and measures against the COVID-19 pandemic to guarantee not only the containment of disease spread but also to fulfill its responsibility as a state toward its people and their safety [14].

### Immediate Response to Contain Disease Spread

One of the first steps taken by the government was to develop functional emergency operations centers and to detect the route of disease spread in Pakistan. The origin of the virus was the first question; hence, detailed history-taking of patients was crucial not only in understanding the outbreak but also in determining the contacts of patients with other people in the community [15]. This helped in cordoning off areas or home-bounding people who came in close contact with a patient with COVID-19. In addition to this, patients with a recent international travel history were monitored closely. This made sense because many cases and massive spread was reported in the countries neighboring Pakistan [15-17].

### Containment Measures

Once primary and secondary contact-tracing was delineated, the foremost step taken by the government was to control the borders [18]. This was a crucial decision, owing to the consideration of a large number of Pakistani students and pilgrims studying in and travelling from China, Iran, and Europe. The government gained the confidence of the affected individuals and their families. It was almost unfeasible to restrict such individuals outside the country because of the strong public response; nonetheless, it was necessary if the spread of the virus was to be controlled quickly. To tackle this problem, the government took the initiative of designating quarantine houses near borders and airports to isolate people entering Pakistan for a short period to make sure they were not infected before they moved out in the community [19,20].

### Border Control

The WHO reported that the number of new cases increased by the minute, and disease spread was now not only limited to people who had a recent travel history in the regions highly affected by the pandemic. Disease spread within the community was alarming and called for drastic steps to be taken not only by local governments but also by countries and states at large. All necessary services and measures are still being used in maximum capacity till date to ensure the safety of people’s lives in the country. Since all cases initially had a history of recent travel, it was speculated that transmissions were imported from outside of the country. Therefore, travel restrictions were imposed to limit the spread of virus from other countries to Pakistan [21].

### Quarantine Houses

After the borders were contained, it was important for the government to provide a solution to all individuals stuck at the borders to enter the country without imposing a threat to the rest of the community [20]. It was crucial to quarantine the people at a specific location and either have them tested or wait for at least 2 weeks to ensure that they were not infected with SARS-CoV-2 before they travelled to their hometowns. People who did not show any symptoms after being quarantined for a certain duration could go to their cities and notify the authorities in case of any signs and symptoms after leaving the quarantine homes [19,20]. The development of these shelters was an economically and strategically massive task for the government. More than 3000 pilgrims arrived from Iran in the first week of March 2020 alone and were housed at quarantine shelters in Taftan and Chaman [22,23].

Toward the end of March 2020, the government decided to relocate the pilgrims to their respective provinces where quarantine centers were set up. Most news outlets and social media users condemned this step taken by the government. Many problems were faced by the pilgrims and other people who were quarantined at these centers, including included small, cramped spaces for people to live in, unhygienic conditions, shortage of food, water, medication, and unavailability of physicians [23].

### Country-Wide Lockdown

Many other steps were taken by the government to tackle disease spread and to minimize the damage caused by the pandemic in Pakistan [24,25].

One of the first steps that the government of Pakistan took to limit the spread of the virus within the community was to impose well-planned lockdowns in all major cities [20,26]. Lockdowns were imposed during different hours in different regions, and most of the public spaces were closed off except for grocery stores, pharmacies, and vegetable and fruit shops. All the eateries, parks, wedding halls, schools, and offices were closed until further notice by the federal government [27]. This led to retaliation from the provincial governments and opposition as it posed a great economic threat to the country’s daily-wage workers and to the low-income population; however, this was a necessary measure to curtail disease spread. Another step that the government took, and faced major opposition, was the closure of prayers at mosques, including Friday prayers [26,28,29].

### Cordoning Off Areas

When reports of virus transmission started emerging, especially in the federal capital of Islamabad, the government took the initiative of sealing off areas that reported infections. According to a notification issued by the District Magistrate Islamabad, the city administration decided to cordon off areas to ensure public safety after the number of infections increased [30]. Samples were tested by the National Institute of Health, Islamabad, and analyzed by epidemiologists of the deputy commissioner of the COVID-19 Nerve Centre after which the notification was issued. This helped in not only curbing the spread of the infection but also in contact-tracing and further testing of the public.

### Testing and Contact Tracing

The country’s testing capacity was limited during the early months of the pandemic, and while high-income countries were conducting large-scale randomized tests to estimate the actual number of confirmed cases, Pakistan was forced to carry out priority-based testing and rely on the enforcement of strict quarantine and isolation strategies to contain the pandemic [31]. Contact-tracing, however, was an effective strategy that not only helped limit the spread of the virus but also helped predict its route through different regions of the country and across different age groups. Nevertheless, since large-scale testing was crucial to assess the severity of the pandemic, the testing capacity of laboratories and the availability of testing kits was gradually increased by the government, and in June 2020 up to 30,000 tests were conducted daily to ascertain the pace of spread and to formulate future strategies accordingly [32]. Both these strategies provided valuable insights on the differences in the clinical manifestation of COVID-19 in people with different demographic and health backgrounds.

### Field Epidemiology Laboratory Training Program

The Training Programs in Epidemiology and Public Health Interventions Network is a network of 75 field epidemiology training programs, which operate in >100 countries including Pakistan. After the WHO declared COVID-19 a public health emergency of international concern, alumni from the Field Epidemiology Training Program implemented standard operating procedures (SOPs) for COVID-19 screening at international airports in Pakistan. They also designed and implemented a real-time data entry system to screen travelers from high-risk countries [33].

### Implementation of SOPs: Masks, Sanitization, and Social Distancing

SOPs were devised for the public and were meant to be strictly followed in public areas. These included guidelines on social distancing; that is, avoiding crowded areas, maintaining a physical distance of 3 feet, wearing masks, maintaining hand hygiene, sanitizing frequently touched surfaces and areas, and following general hygiene rules such as avoiding touching the face, nose, or eyes, and coughing, or sneezing in the elbow or a paper napkin instead of the hands. The authorities started taking disciplinary action against those who violated the SOPs at public places in various parts of the country in accordance with the recommendations of the National Command and Control Centre of Pakistan. The focus of the National Command and Control Centre was on SOPs, compliance, strict administrative actions being implemented, and enforcement of various strands of the track, trace, and quarantine strategy [34].

### Initiation of Awareness Campaigns: Role of Community Health workers

Many campaigns were initiated by both local and federal governments in the interest of the general population to spread awareness about the risks, signs, and symptoms of COVID-19 [35]. Pakistan’s extensive polio vaccination program, consisting of more than 265,000 community health workers and vaccinators, was mobilized with the help of the WHO [36]. This not only helped provide infrastructure to track and trace cases early during the epidemic but also helped spread awareness in the remote, underdeveloped rural regions of Pakistan. Another vital step was taken to spread awareness to the masses, where text messages were sent by the government of Pakistan on all mobile networks [37]. The daily reminders on following SOPs helped tackle those who did not take the necessary precautions and were unaware of the aforementioned information, and the imposition of fines and charges for noncompliance made risk and awareness campaigns a nationwide success [35,37].

Recorded voice messages in various local languages including Urdu, Pashto, and Sindhi, which warned against the risks of COVID-19, its spread, and its complications, and general awareness regarding the SOPs to help control its spread, were used as caller tunes before every phone call. The recorded messages were changed in accordance with the situation and ranged from guidelines on SOPs, warning noncompliers, and even congratulating efforts after successfully controlling disease spread during August 2020 [37].

### Economic Measures

On the emergence of COVID-19 in Pakistan, the entire system faced various problems owing to the limitations of the health care system, poor infrastructure, uneven access to health care, resistance from various social, political, cultural, and religious groups, political instability, economic fragilities, and mistrust among the public. Data of a web-based survey conducted by the Small and Medium Enterprises Development Authority from April 3-14, 2020, among 920 businesses revealed insufficient revenue generation, losses, and difficulty in survival among businesses [38]. Pakistan launched various schemes to tackle the economic crisis faced by many individuals during the pandemic. On May 2, 2020, Prime Minister Imran Khan launched a relief scheme for people who lost their jobs or whose source of income has been compromised owing to the lockdown. He launched a cash assistance program through the Ehsaas Cash Programme to support unemployed individuals [39].

After lifting the lockdown in some sectors, the government allowed construction and daily-wage workers to resume working while dutifully following the SOPs and taking necessary precautions. This helped ease some of the economic burdens of the government, especially in providing rations and relief packages for the daily-wage workers [40].

Furthermore, the government also requested people who had been diagnosed with mild or asymptomatic COVID-19 to quarantine at home as some of them did not required hospital care; this helped curb the patient influx in hospitals and at diagnostic centers, thus easing the burden on health workers and medical practitioners [41].

### Production of Ventilators

One of the largest concerns for the ministry of health and health departments in Pakistan and worldwide is coping with the continuously increasing demand of ventilators as the virus spreads and the number of cases increases. The shortage of ventilators is a major issue faced by Pakistan, especially because all the major medical equipment are imported and not produced locally. To tackle this problem, the National Radio and Telecommunication Corporation produced its first ventilator locally within a few months of the onset of the pandemic [42]. The National Radio and Telecommunications Corporation initially offered cost-free repairs for almost 109 ventilators throughout the country and later designed and produced its own ventilators. Initially, 8 ventilators were produced and handed over to the National Disaster Management Authority after which the prime minister formally inaugurated a facility for large-scale production of ventilators within Pakistan [43].

As a result of all the efforts made by the government of Pakistan, 6 months after reporting its first case, active cases in Pakistan are continuing to steadily decrease, with the number of deaths recorded in a day now often down to single-digit numbers. The country has had 312,263 confirmed cases of as on October 1, 2020, with 6479 COVID-19–related deaths, according to the official data [44]. Save for single-day glitches, active cases have been progressively declining since peaking in June 2020, currently standing at 8903, their lowest level since late April 2020.

## Success Stories: Pakistan’s Population Coming Together to Combat COVID-19

Pakistan is currently faced with one of its toughest challenges since its establishment as an independent nation, and while COVID-19 has severely disrupted routines and led to intense fear among the public, efforts are still being made to bring together the expertise and knowledge of individuals from various fields to ensure combating this pandemic together. In addition to the government’s efforts, many other organizations and individuals came forward to help against the pandemic in various capacities. From using social media to campaign for blood donations for patients with thalassemia nationwide to arranging food supplies and relief packages for those severely affected by the pandemic, the local people of Pakistan came forward and helped fellow citizens.

The Human Development Foundation Pakistan is one of the oldest nonprofit organizations in the country, and it estimated the provision and distribution of more than 14,000 ration packages containing food and medical supplies to the people of Karachi [45].

### Al-Khidmat Foundation Pakistan

Al-Khidmat Foundation Pakistan is one of the leading nonprofit foundations operating in Pakistan for the past 30 years. During the onset of the pandemic, it played a vital role in charity work including awareness campaigns, providing preventive equipment such as soap, sanitizers, masks, and relevant reading material to the general public, providing its services to government institutions including hospitals, Aghosh homes, ambulances, and trained volunteers. Moreover, it carried out food drives, provided COVID-19 test facilities and antibody tests, and arranged for protective equipment to distribute among physicians and medical staff on the frontline [46]. Al-Khidmat Foundation Pakistan also played a vital role in arranging free plasma for patients with COVID-19 [47].

### Corona Recovered Warriors

The Facebook page titled “Corona Recovered Warriors” provided hope during the grim period of disease spread in Pakistan. Created by musician Zoraiz Riaz with the aim to help coordinate convalescent plasma donations for people with COVID-19 in Pakistan, this group quickly gained popularity and had 320,000 members in just 1 month, needing a team of 33 volunteers to manage the posts. People looking for plasma donors, medical supplies, oxygen cylinders, ventilators, injection drugs or other drugs, and leads on hospitals accepting new admittees posted all their queries on this group, and thousands of people came forward and offered help in all capacities to those in need [48]. The group then started organizing donation and food drives, providing personal protective equipment (PPE) to health care workers and delivering medical supplies to desperate families of patients with COVID-19 [49].

### Plasma Trials by Medical Professionals

In desperate times when everyone is seeking any cure to tackle the virus, many unapproved and untested remedies were used even by health care professionals to treat individuals with COVID-19. The most popular one was the use of plasma of a recovered patient to treat patients with COVID-19. Dr Shahid Junejo, a senior superintendent at a civil hospital, tested plasma therapy on a patient in Hyderabad. According to him, the decision for the trial was taken after consultation with the vice chancellor of the Liaquat University of Medical and Health Sciences Jamshoro, Prof Bikha Ram Devrajani [50]. Passive immunization is an old procedure used in the absence of a vaccine to treat infectious diseases; hence, the treatment was administered to many patients throughout the country without any strong evidence of its ability to neutralize the virus [51]. Even though the Ministry of National Health Services declared that plasma therapy is not a cure for COVID-19 as it is still undergoing a clinical trial on a global scale, there is still a high demand of plasma donors, and as of July 2020 approximately 750 donors were connected with critical patients through the Corona Recovered Warriors Facebook page alone.

### Availability of Scholars Against COVID-19 Pakistan

Scholars Against COVID-19 is a platform of over 3000 young scholars and researchers nationwide coming together as volunteers to assist the government and people through donations of equipment from laboratories and universities to scale up testing and experimentation related to the diagnosis and treatment of COVID-19 [52]. Their aim is to bridge the disconnect among various sectors in Pakistan, which makes executing such ideas challenging.

### Student Taskforce Against COVID-19

Student Taskforce Against COVID-19 is a group of final-year medical students from Aga Khan University Hospital who conceived the idea of creating a helpline for families and patients affected by COVID-19 who have been looking for guidelines and help [53]. The taskforce is not only managing the helpline but also assisting the Aga Khan University Hospital and the Sindh government in contact-tracing and helping the Pakistan Medical Association in identifying and providing volunteers to help at Karachi's Expo Centre isolation ward for patients with COVID-19 [54].

### First Response Initiative of Pakistan

Over 400 medical students have come together through the Combat Corona campaign and are aiming to collect and provide PPE to health care workers. They have targeted hospitals in Karachi needing PPE and have collected hundreds of equipment for distribution through donations in all major hospitals of the city [54].

### Pakistan Against COVID-19 Volunteers Group

Pakistan Against COVID-19 Volunteers is a group of physicians and other professionals who have collaborated and aim to enable connections between manufacturers and suppliers of PPE, carry out innovation and experimentation for designing and manufacturing ventilators through 3D printing technology, and develop noncontact thermometers locally [55,56].

### Kwickdoctor.com

Kwickdoctor.com has been developed by 40-year-old information technology expert Salman Khan. He aims to connect the public to consultants, physicians, pharmacies, and laboratories and even delivers prescriptions at the doorstep. With the pandemic spreading, the need to avoid contact has increased and digital care is the need of the hour [57].

## Discussion

### Principal Findings

COVID-19, after being first reported in December 2019, is still swiftly spreading worldwide. Within 10 months, the mortality and morbidity rates have approached unexpected levels. Scientists, researchers, and clinicians have worked together with engineers to develop treatments, diagnostic kits, and vaccines to prevent this infection from spreading further; however, a third wave of COVID-19 is currently underway worldwide with numerous mutant strains. These mutations have rendered this virus either more virulent or resistant to previously used medications [58]. The second wave has been feared as the situation was reverting to normalcy and businesses, offices, and schools were reopening in late September or early October of 2020. The government of Pakistan started tackling COVID-19 on the basis of the experience of other countries as the disease approached Pakistan after having affected many other countries. Starting from preparing special wards, using all resources including polio and dengue teams and wards, respectively, preparing appropriate SOPs, conveying awareness messages to everyone through the television or mobile phones, updating everyone through special mobile apps and websites, showing hotspots, sharing the economic burden with the weak, and ending with smart lockdowns were some of the most impressive measures to handle the pandemic. The people of Pakistan also supported the government during this time, which is one of the most prominent reasons Pakistan overcame the first 2 waves with minimal morbidities compared to other countries.

### Conclusions

Immunization and treatment of COVID-19 may still be questionable, but precautions and SOPs have undoubtedly been set by many. Technology has played its part in spreading awareness, but Pakistan is currently undergoing a third wave of infections. However, if precautions are taken and all SOPs are followed, the entire community can be rescued and the risk of reinfection and further waves would decline immediately. This is a situation where everyone has a responsibility toward the community and must take steps to minimize the risk of further disease spread. Pakistan has shown tremendous potential in public health, and different government and nongovernment organizations can collaborate to address the challenges through the engagement of society and the community along with the introduction of new policies.

## Abbreviations

PPE: personal protective equipment
SOP: standard operating procedure
WHO: World Health Organization
